# Efficacy of Isometric Exercises and Somatosensory Training for Pain, Proprioception, and Balance in Runners with Patellofemoral Pain Syndrome

**DOI:** 10.7759/cureus.56163

**Published:** 2024-03-14

**Authors:** Shraddha S Kochar, Tejaswini Fating, Shubhangi Patil

**Affiliations:** 1 Community Health Physiotherapy, Ravi Nair Physiotherapy College, Datta Meghe Institute of Higher Education and Research, Wardha, IND

**Keywords:** proprioception, pain, balance, somatosensory training, isometric exercises, runners, physiotherapy, anterior knee pain, patellofemoral pain syndrome

## Abstract

Background

A significant cause of knee pain is patellofemoral pain syndrome (PFPS). Young adults are the most common population to be impacted, and this condition appears to affect both sexes. Patellofemoral joint (PFJ) compression, which is felt around the patella during any physical or athletic activity, usually causes patients to experience pain in the anterior part of the knee. Physiotherapy is essential for patients suffering from this illness, as it can improve their everyday activities and ability to return to their sport.

Methodology

The study's main goal was to evaluate the effectiveness of somatosensory training and isometric exercises for pain, proprioception, and balance in runners with PFPS. Before- and after-test approaches were used in the investigation. Eighty-five people made up the study, with the inclusion and exclusion criteria used to determine eligibility. Isometric exercises and somatosensory training were given to every individual; the group was not randomly assigned. The patient's diagnosis was made using the patellar grind test. Participants received 30-45 minutes of isometric exercises and 15 minutes of somatosensory training every four days. The visual analog scale, joint position sense test, and Y-balance test were taken as outcome measures to measure PFPS before and after the intervention.

Results

The result revealed significant (p=0.0001) improvement in PFPS following the intervention. Both the isometric exercises and somatosensory training were found to be significant in reducing the intensity of the pain and improving the proprioception and balance of the individuals.

Conclusion

Both treatment approaches were beneficial in lowering pain in the joints, developing balance, and helping the patient perceive the position of the joint. Individuals can use both therapy methods to improve their running abilities, and they should become ingrained in daily practice.

## Introduction

Running, descending stairs, or crouching are a few instances that burden the knee during flexion and are associated with patellofemoral pain syndrome (PFPS), which appears as diffuse retropatellar or peripatellar complaints [1]. Persistent and long-lasting symptoms can hinder one's ability to engage in physical activity, sporting events, or employment [2]. Significantly, patellofemoral pain may appear before the emergence of incurable patellofemoral osteoarthritis [3]. PFPS, a condition known as anterior knee pain (AKP), is a pain that originates in the patellofemoral joint (PFJ) [4]. The acronym "PFPS" is frequently employed when referring to the condition of pain in the anterior knee, which incorporates all problems pertaining to the anterior area of the knee [5]. Around 25% of all current injuries due to running in the field of sports medicine are caused by PFPS [6]. The PFPS mainly influences young women because of the anatomic, hormonal factors, and knee laxity, which are more commonly found in women and do not induce any significant disease-related symptoms such as an enlarged quadriceps (Q) angle [7]. PFPS is the most typical etiology of knee pain, with prevalence rates ranging from 15% to 45%. The literature has claimed that muscle imbalance is a critical determinant of the observed patellar maltracking that triggers patellofemoral pain [2]. This asymmetry comprises decreased quadriceps muscle strength and volume, notably in the vastus medialis oblique (VMO) muscle [2]. Anatomical irregularities, poor quadriceps or iliotibial band versatility, altered lower-limb biomechanics (whether static or dynamic), muscle disorder (which includes quadriceps weakness or inappropriate firing pattern), patellar hypermobility, surgical intervention, tight lateral structures (e.g., the lateral retinaculum and iliotibial band), training inconsistencies or overloading, and trauma are all major risk factor for PFPS [8].

Many musculoskeletal injuries are commonly treated using manual therapy [9]. The conservative approach to treatment includes patient education, manual therapy, neuromuscular electric stimulation of the quadriceps, therapeutic ultrasound, activity modification, biofeedback, exercises to ramp up the activity of the VMO muscle, stretches for tight structures, muscle strengthening exercises for the lower limbs, proximal stabilization, bracing, foot orthotic devices, and taping methodologies [10]. The foremost choice of management for PFPS is prescribed to be rehabilitative exercise therapy [8]. Individuals experiencing PFPS are advised a number of exercises according to three distinct criteria: muscle activity (isometric, eccentric, or concentric), joint movement (dynamic, static, or isometric), and reaction forces (open or closed kinetic chain) [8]. While recommending performing suitable exercises can frequently assist individuals with PFPS to achieve better results, several PFPS patients have a range of difficulties in their central and peripheral pain mechanisms [11].

Research has demonstrated that isometric exercises can minimize pain in the anterior aspect of the knee, promote functional capacity, and enhance the PFJ area of contact [12]. Some pilates exercises, such as the hundred and single-leg circles, incorporate specific isometric exercise aspects and have been evaluated for their efficacy in treating a variety of knee problems [13]. One of the most appealing therapeutic interventions to manage PFPS has also been recognized as isometric exercise [14]. It has additionally been observed that patellofemoral pain is connected with somatosensory abnormalities [12]. According to studies, individuals with PFPS have weaker proprioception and postural balancing abilities than those without PFPS [12]. One of the most debatable non-pharmacological therapeutic approaches for PFPS is exercise [8].

Since PFPS ranks as one of the most prevalent musculoskeletal-related conditions, it impacts a large number of runners around the globe. Physiotherapy serves a very significant part in restoring the patient's mobility, decreasing pain, improving muscular flexibility and strength, enhancing performance, and boosting the quality of muscle, which has expanded the demand for physiotherapy. As isometric exercises reduce patellofemoral pain, raise functional ability, and improve the PFJ area of contact, studies have found that somatosensory training, a new form of training, is an effective treatment for PFPS to improve proprioception and balance. Running enthusiasts make up the participants in this research, as there has not been a study on isometric exercises and somatosensory training in runners yet. This study's primary goal is to determine the efficacy of isometric exercises and somatosensory training for pain, proprioception, and balance in runners with PFPS.

## Materials and methods

Before the commencement of the study, clearance was obtained from the institutional ethical committee of Datta Meghe Institute of Higher Education and Research, Sawangi, Maharashtra, India (Ethical authorization number: DMIHER(DU)/IEC/2023/1000). Researchers conducted this interventional from August 2023 to February 2024. Participants with PFPS between the ages of 18 to 40 years made up the study's subjects. To improve pain, proprioception, and balance in runners with PFPS, the study intended to assess the effectiveness of isometric exercises and somatosensory training.

Using data from Sahu's survey on the prevalence of AKP, the total number of patients participating in the study was calculated using Cochran's formula [4]. Male and female runners between the ages of 18 to 40 years who were willing to engage in the study were included in our research. Participants who were diagnosed with jumper’s knee, had a history of prior knee surgery, or participated in any sport other than running were excluded. Those who were willing to participate filled out written consent forms and signed them after being informed of the study's goals and methodology.

Data collection tool and technique

A total of 85 PFPS patients were enrolled in the study. Each participant was asked to provide informed permission and was explained about the study. All interested individuals were evaluated based on inclusion and exclusion criteria. Before any interventions were administered to the patients who had been chosen, they were extensively examined. If the patient satisfied the eligibility requirements, they were included in the study. The study interventions were carried out by a faculty physiotherapist to whom the concerned intern was assisting. A pre- and post-experimental design was used for the study, with all the individuals receiving the isometric exercises and somatosensory training. The individuals were diagnosed based on the patellar grind test (Clarke’s sign). The patellar grind test or Clarke's sign determines if patellofemoral dysfunction is present. The patient was lying on their back or long sitting with the affected knee extended. The therapist applies pressure while placing her hand's web space above the patella. The quadriceps muscle was contracted gradually and softly by the patient. Pain in the PFJ was the positive indicator for this test.

Pre- and post-intervention evaluations were conducted for all subjects using the visual analog scale (VAS), joint position sense test, and Y-balance test (YBT). Each patient was required to complete four weeks of rehabilitation after enrolment in the study, which included, on average, 30-45 minutes of isometric exercises per day for four days per week in addition to 15 minutes of somatosensory training.

Researchers assessed the effects of the interventions by conducting pre- and post-intervention assessments of the subjects using VAS, joint position sense test, and YBT. This rigorous design and evaluation process aims to determine the efficacy of isometric exercises and somatosensory training for pain, proprioception, and balance in runners with PFPS.

Interventions

Isometric Exercises

All the runners with PFPS underwent an intervention program on average of 30-45 minutes per day for four days per week for four weeks of the treatment protocol. Before treatment, a thorough physiotherapy assessment was conducted, and the outcome measures were also taken. The isometric exercises were done for both the knee and the hip muscles. The treatment included static quadriceps, static hamstrings, hip flexors, hip abductors, and hip adductor isometric exercises. All the exercises were repeated 20 times in the treatment session. Table 1 shows the isometric exercises that were given to the patients.

**Table 1:** Isometric exercises that were given to the patients

Exercise	Procedure	Dosage
Static quadriceps	The patient was asked to be in supine lying position with the foam roll placed underneath the knee. Then the patient was told to try to push down the foam ball with their knee while contracting the quadriceps muscle and then hold it for 3 to 5 seconds.	10 repetitions x 2 set
Static hamstrings	The patient was brought into a supine lying position and instructed to squeeze their hamstrings for 3 to 5 seconds by pressing their heel down into the surface underneath them.	
Hip flexors isometric exercise	The patient is placed in a supine lying position with their hips and knees in a 90-degree flexion position. The patient is then asked to attempt to push their leg upward against the hand while the therapist applies resistance in the opposite direction and the patient holds their leg in place for 3 to 5 seconds.	
Hip abductors isometric exercise	The patient was requested to push their legs apart, just like they were trying to open them while lying with their knees bent at a 90-degree angle and a resistance band wrapped around their thighs. For 3 to 5 seconds, they were instructed to maintain their position. Care was taken to ensure the person exercising did not experience pain or noticeable motions.	
Hip adductor isometric exercise	With their knees bent to a 90-degree angle, the patient was positioned in a long sitting position with their feet flat on the surface beneath them. Following 5 seconds of pressure application, the participant was instructed to ease up and release the pressure on the pad.	

Somatosensory Training

Every runner completed a 15-minute program four days a week for four weeks. The program included weight-bearing exercises focused on postural balance, proprioceptive stimulation, and muscle strengthening. Both hard and unstable surfaces were used for the exercises. These exercises were done with closed eyes after the exercise was done with open eyes. Every exercise was carried out according to the level of difficulty. Table 2 shows the protocol that was given for somatosensory training to the individuals.

**Table 2:** Somatosensory training which was given to the patient reps, repetitions

Exercise	Procedure	Dosage
Side stepping	After instructing the patient to walk laterally, separating, and re-joining their feet, they are advised to assume a partial squatting stance with their feet together.	10 reps x 2 sets
Lateral lunge	Maintaining a slight distance between the feet while they stand. Step to the side and then bend the knee to make a lunge. Maintaining alignment of the knees with the toes allows the bottom to descend toward the floor while the knee bends. With each knee bend, the patient was instructed to raise their arms forward to provide a counterbalance.	3 seconds of hold with 10 reps x 2 sets
Standing on one leg	After assuming a wide base of support, the participants were instructed to flex their hips and knees to a 90-degree angle and hold that posture for 3 to 5 seconds while keeping their balance.	10 reps x 2 sets
Standing on one leg with heel raised	The patient was instructed to stand on one leg, elevate their heel off the ground, and stand on the tips of their toes.	3 seconds of hold with 10 reps x 2 sets
Posterior lunge	With the feet hip-width apart, start standing. To lower their body weight toward the ground, the patient was directed to step back with one leg while bending both knees, and at the same time, try to maintain an upright chest. With the thigh still perpendicular to the floor, the back knee should descend straight to the floor.	3 seconds of hold with 10 reps x 2 sets
Lateral step-up	Moving to the side and onto the elevated surface they were standing next to. On the elevated surface, both feet should be in contact. Then, descend to the floor and move in the direction of the side from where they began.	2 seconds of hold with 10 reps x 2 sets
Air squatting	Toes pointed forward and slightly turned out, feet shoulder-width apart. Bend at the hips and knees, lowering the buttocks toward the floor while maintaining a straight back. Then let the patient's arm come forward as they descend, then allowing them to straighten their arms and raise them back up. It is appropriate for the buttocks to drop below the feet in a chair-like position. The patient was instructed to highlight the weight that passed through their heels.	2 seconds of hold with 5 reps x 2 sets

Outcome measures

Visual Analog Scale

The VAS is a frequently used assessment tool for pain intensity in rehabilitation, which has been demonstrated to be reliable. It is a unidimensional measure to assess pain. A 10-cm-long, horizontal, straight line extends from left (0) to the right (10) with its ends marked as 0, which indicates no pain, and 10, which indicates extremely severe pain. This is also used for pre- and post-intervention [15].

Joint Position Sense Test

The joint position sense test is assessed by reproducing active and passive joint positions. The therapist places the limb at some preset angle and holds it there for 10 seconds to allow the individual to mentally process the angle the then limb is taken to a neutral position, and the person is asked to actively or passively take their limb so the same degree by keeping the eyes closed to avoid any visual cues [16].

Y- Balance Test

Dynamic balance can be measured with the help of the simple YBT. To complete the YBT, a person must balance on one lower limb and simultaneously extend their other leg as far as they can in the anterior, posterolateral, and posteromedial directions. As a result, this examination assesses the participant's balance, strength, and stability in multiple dimensions [17].

## Results

Statistical analysis was conducted using descriptive and inferential statistics using Student’s paired t test. Software used in the analysis was SPSS Version 27.0 (IBM Corp., Armonk, NY), and p<0.05 was considered as level of significance. Tables 3, 4 show the distribution of patients according to their age in years and the distribution of patients according to their gender, respectively.

**Table 3:** Distribution of patients according to their age SD, standard deviation

Age group (in years)	Number of patients	Percentage
18-27	53	62.35
28-37	26	30.59
38-47	6	7.06
Total	85	100
Mean±SD	25.49±6.60 (18-40 years)	

**Table 4:** Distribution of patients according to their gender

Gender	Number of patients	Percentage
Male	46	54.12
Female	39	45.88
Total	85	100

Table 5 shows a comparison of VAS scores pre- and post-treatment. The mean VAS scores pre- and post-treatment were 7.51 and 4.64, respectively. Using the Student’s paired t-test, a statistically significant difference was found in VAS scores pre- and post-treatment, with the t-value being 37.75 and the p-value being 0.0001. Pre-intervention, the mean score was calculated, which was 7.51; again, it was calculated after the whole treatment duration. The results revealed a significant decrease in the mean score to 4.64. The standard deviation of the pre- and post-treatment was 0.89 and 0.83, respectively. The considerable reduction in the mean score signifies the notable impact of the intervention's effect on the individual's performance. When comparing the post-test findings to the pre-test, the reduction in standard deviation corresponds to improved consistency and precision.

**Table 5:** Comparison of VAS scores pre- and post-treatment VAS, visual analog scale

Test	Mean	N	Standard deviation	Standard error mean	Mean difference	Values
Pre-treatment	7.51	85	0.89	0.09	2.87±0.70	t-value = 37.75, p-value = 0.0001, significant
Post-treatment	4.64	85	0.83	0.09		

Table 6 shows a comparison of joint position sense test scores pre- and post-treatment.

**Table 6:** Comparison of joint position sense test pre- and post-treatment

Test	Mean	N	Standard deviation	Standard error mean	Mean difference	Values
Pre-treatment	9.72	85	2.41	0.26	7.29±2.84	t-value = 23.60, p-value = 0.0001, significant
Post-treatment	2.43	85	1.25	0.13		

Table 7 shows a comparison of Y-balance test scores pre- and post-treatment.

**Table 7:** Comparison of Y-balance test pre- and post-treatment

Test	Mean	N	Standard deviation	Standard error mean	Mean difference	Values
Pre-treatment	47.04	85	5.06	0.54	26.65±5.63	t-value= 43.65, p-value= 0.0001, significant
Post-treatment	73.70	85	6.09	0.66		

## Discussion

The purpose of the present research was to examine how somatosensory training intervention programs and isometric exercises affect runners with PFPS in regard to pain, proprioception, and balance. There have been previous research investigations on PFPS, which had favorable results when treated with physiotherapy. Exercises for rehabilitation have been recommended as the cornerstone of PFPS management with the goal to ease pain, improve the patient's ability to perceive joint position, and maintain balance. Gaining strength in the muscles of the lower part of the body as well as other functional skills such as proprioception and postural balance seem to be beneficial in helping runners’ performance and avoiding accidents.

In our study, a total of 85 runners with PFPS who met the inclusion and exclusion criteria were selected for interventional analysis. The individuals were treated using isometric exercises, which included static quadriceps, static hamstrings, hip flexors isometric exercise, hip abductors isometric exercise, and hip adductor isometric exercise, in addition to somatosensory training, which was used to improve proprioception and balance. This training included side stepping, lateral lunge, standing on one leg, standing on one leg with heel raised, posterior lunge, lateral step-up, and air squatting. In this study, considering the level of pain, ability to sense the joint position, and ability to balance, which were associated with PFPS, all the individuals showed a significant pre- and post-difference. Three outcome measures were used to assess the patient’s pre- and post-rehabilitation improvement. These were evaluated on day 1 and the last day of the treatment.

Steinberg et al. investigated the effect of isometric exercises and somatosensory training as intervention programs for PFPS in young dancers. The total sample size of the study was 98, with the age group of 12-15 years, and the individuals were randomly divided into three groups. The rehabilitation program was conducted for 12 weeks. In conclusion, the study showed that isometric exercises and somatosensory training were both effective in reducing pain and improving the proprioception and balance of young dancers [12]. We included this in our study and found the results to be effective on the individuals suffering from PFPS.

A study was conducted by Mason et al. to examine the effect of patellar taping, quadriceps strengthening, and quadriceps stretching prescribed separately or combined on PFPS. Each of these exercises was given in isolation for one week and in combination for one week. The study was designed via a prospective, double-blind, randomized control study, which included 41 subjects divided into four different groups. Seven pre- and post-treatment measures were included. Results showed significant changes over time in two out of seven measures for the taping group, five out of seven for the strengthening group, five out of seven for the stretching group, and none in the control group. When all of them were combined for one week, all seven measures improved significantly. In conclusion, quadriceps strengthening and quadriceps stretching were found to be more effective than taping in isolation. A combination of these treatments was recommended as an initial approach to PFPS [18].

The research was conducted by Gilson et al. whose objective was to see the effectiveness of hip-strengthening exercises in managing PFPS in females. The study showed that both the hip and knee exercises must be included in the patient's treatment regime rather than only including hip strengthening exercises. Combined hip and knee exercises were seen to be more effective than only including the hip exercises [19]. Our study also included both hip and knee exercises, which were significant in improving the patient's pain.

A study was conducted by Vinaya Kumar et al. with the objective of assessing and comparing the effectiveness of neuromuscular electrical stimulation application (NMES), quadriceps strengthening, and, in combination, the quadriceps muscle architecture and functional capacity in patients with PFPS. This study included 124 participants in the age group of 18-40 years, who were randomly allocated into four different groups. The four groups were as follows: group A consisting of NMES, group B consisting of quadriceps strengthening, group C consisting of both NMES and quadriceps strengthening, and group D was the control group. The treatment was given for 10 weeks. In conclusion, the combination group showed significant improvement when compared to the quadriceps strengthening group followed by NMES. The control group did not show any improvement [20].

Limitations

The limitations of the study are that the trial lasted for only four weeks, there was no long-term follow-up with the individuals, the lack of strength evaluation, and lack of morphological and histological evaluation. More interventional studies must be conducted on the intervention applied in this study.

## Conclusions

The functional capacities of runners with PFPS were strengthened by isometric exercises and somatosensory training, which significantly reduced clinical symptoms, including pain, and promoted proprioception and balance in the individual. Therefore, both the physiotherapeutic interventions were observed to be effective and significant in runners with PFPS. Further research needs to be conducted to find the best training schedule for preventing and treating PFPS in runners.
